# The UK cellular microbiology network: Exploring the host‐bacterial interface

**DOI:** 10.1111/cmi.13081

**Published:** 2019-07-17

**Authors:** Jennifer L. Rohn, Serge Mostowy, Jason S. King, Meera Unnikrishnan, Maximiliano G. Gutierrez

**Affiliations:** ^1^ Department of Renal Medicine, Division of Medicine University College London London UK; ^2^ Department of Infection Biology The London School of Hygiene and Tropical Medicine London UK; ^3^ Department of Biomedical Science University of Sheffield Sheffield UK; ^4^ Division of Biomedical Sciences University of Warwick Coventry UK; ^5^ Host‐Pathogen Interactions in Tuberculosis Laboratory The Francis Crick Institute London UK

## Abstract

The UK Cellular Microbiology Network held its inaugural conference in February 2019. This stimulating day of scientific exchange will be the first of many, its organisers hope.

## 1 ARTICLE

In an era of vast conferences with thousands of participants, where poster sessions may be held in halls the size of aircraft carriers, scientists often find solace in smaller meetings. These allow better opportunities for personal connections, but also permit focusing on topics that seldom get much airplay in modern, multi‐stream mega‐symposia. They can also serve to create and consolidate valuable regional or national networks.

The cellular microbiology of bacterial pathogens is one such niche topic within the greater umbrella of microbiology, with its exotic array of viral, fungal, and bacterial microbes jockeying for position, alongside the competing interests of the immunological response. In an era of increasing antibiotic resistance, furthering our understanding of how prokaryotes interact with their host cells, and developing novel ways to intervene, has never been more important. Despite this imperative, conferences focusing on this topic are rare, and its researchers are often situated in academic departments with different foci, meaning that they can sometimes feel like isolated members of a diaspora and often struggle to meet valuable collaborators.

For this reason, we decided to organise an annual forum in the United Kingdom—dubbed “The UK Cellular Microbiology Network”—devoted to the cell biology of host‐bacterial interactions. Despite the national emphasis, we were keen to welcome any of our geographical neighbours who wished to attend. We wanted to focus specifically on how bacterial pathogens subvert host cellular processes and how the host cell responds. In creating an engaging, affordable, and informal meeting showcasing the work of early‐career researchers, we hoped to encourage the forging of useful collaborations and cross‐fertilisation of diverse expertise and experiences about host cell biology across the wide variety of bacterial pathogens under study.

**Figure 1:**
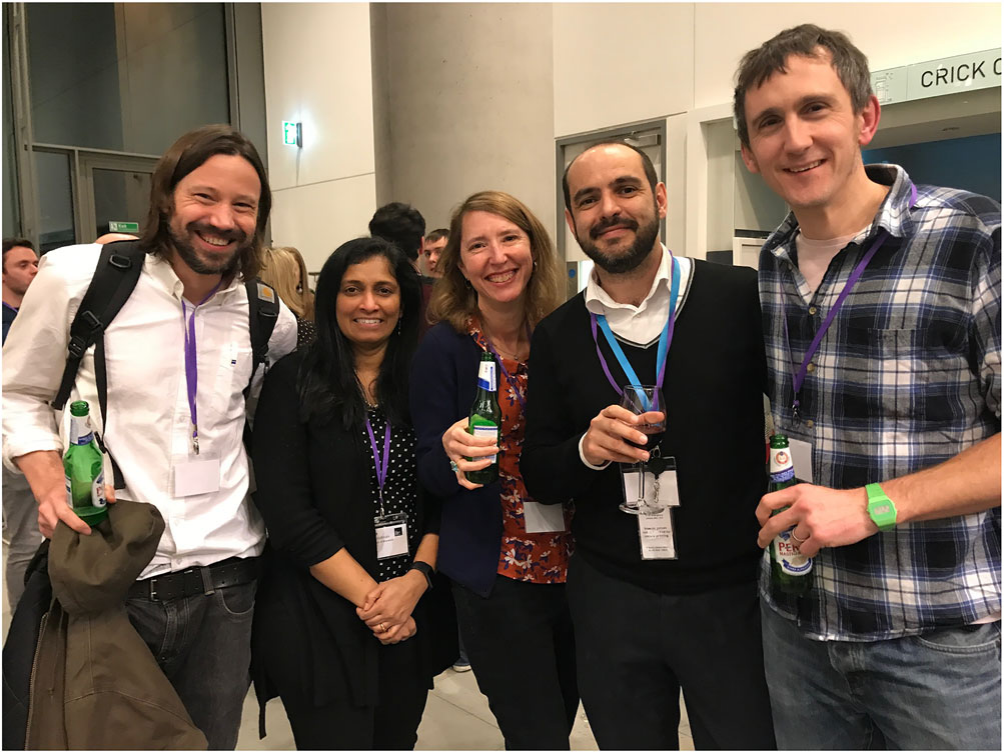
The organisers at the closing reception (from left to right): Serge Motowy, Meera Unnikrishnan, Jenny Rohn, Max Gutierrez and Jason King

Our inaugural one‐day meeting was held at London's Francis Crick Institute on 11 February 2019 (Figure 1). About 140 researchers from across the United Kingdom and a few other European countries gathered in Central London on a cold, sunny day, along with representatives of our generous sponsors, including the Microbiological Society, the Biochemical Society, the British Society for Cell Biology, the *Journal of Cell Science*, Perkin Elmer, Zeiss—and this journal, *Cellular Microbiology*. In addition to the programme of talks, we also had a vibrant poster session and ended the evening with drinks, nibbles, and serious networking in the Crick's lovely gallery space.

The meeting kicked off with the first of our two keynote speakers, Carmen Buchrieser from the Institut Pasteur in Paris. Challenging her audience with the idea that *Legionella* is “the best cell biologist,” she went on to describe some of her team's groundbreaking work exploiting this bacterium to understand host cell function. In the first half of her talk, she demonstrated how the bacterium, once phagocytosed into lysosomes, secretes around 300 proteins to make a replicative vacuole. In the process, it hijacks various host cytoskeletal regulators to fragment mitochondria. This act of sabotage seems to set up a “Warburg‐like” environment of high oxygen consumption and high glycolysis that helps the microbes to replicate. Her team identified a T4SS‐secreted effector of *Legionella* that is implicated in inducing this mitochondrial fragmentation. Most interestingly, this effector encodes protein domains normally only found in eukaryotes. She then showed that such eukaryotic domains are a specific feature of the *Legionella* genomes as she described an exciting comparative functional analysis of 80 *Legionella* genomes spanning 58 species, many of which have acquired the ability to infect eukaryotic cells independently. Indeed, in the process, these species have co‐opted a large number of host genes in order to subvert their hosts, courtesy of the highly conserved type IV secretion system (T4SS).

Secretion—a popular virulence strategy amongst prokaryotes—featured in two additional talks. Focusing on the typhoid toxin produced by the human pathogen *Salmonella* Typhi, Angela Ibler (from Dan Humphreys' lab at the University of Sheffield) demonstrated beautifully how secreted bacterial factors can have an impact on host cell states. Using stunning super‐resolution microscopy images, she showed that the toxin can induce a novel nuclear DNA damage response, called the Response Induced by the bacterial Genotoxin (RING), which was caused by replicative stress resulting from reduced cellular levels of a single‐stranded DNA‐binding protein. Interestingly, this new RING pathway also triggered a transmissible senescence‐like state in cells, which in turn made cells more susceptible to *S*. Typhi infection. This research suggests a new virulence mechanism that may be relevant to chronic *Salmonella* infections. And Angela's talk was so accomplished that she won the prize for best oral presentation—a pair of snazzy binoculars courtesy of Zeiss for whenever she fancies taking a break from the microscope (The other binoculars prize for best poster was scooped up by her labmate Mohamed El Ghazaly, who presented his PhD work on identifying the proteins involved in causing the senescence induced by *S*. Typhi).

Carrying on with the theme that bacterial pathogens employ a number of secretion systems critical for intracellular bacterial survival, Kate Watkins (from Meera Unnikrishnan's group at the University of Warwick) presented her studies on the intriguing type VII bacterial secretion system (T7SS) of the Gram positive pathogen, Staphylococcus aureus. Watkins demonstrated with striking timelapse videomicroscopy images that S. aureus mutants lacking different components of the T7SS were deficient in their ability to escape from macrophages. She showed that T7SS modulated the type of cell death triggered in macrophages, indicating a key role of this system in S. aureus‐macrophage interactions.

Of course, macrophages are a popular host cell for a wide variety of pathogens seeking to gain easy cellular entry and the upper hand against the immune response. Their subversion formed another thread at the meeting, and two talks about the obligate intracellular bacteria Mycobacterium tuberculosis highlighted this theme. Susanne Herbst (Max Gutierrez's lab at the Francis Crick Institute) showed that a kinase commonly associated with Parkinson's disease, LRRK2, has an immune function in macrophages. She showed that cultured primary mouse and human macrophages lacking LRRK2 were able to better control *M. tuberculosis* infection. On the basis of the data she presented, it is tempting to hypothesise that infectious diseases and macrophage responses could play an important role in the pathogenesis of Parkinson's disease. This finding adds to the growing evidence that the immune response contributes to neurodegeneration, which may open the door to innovative new therapies.

It is known that immune cell metabolism is key for the establishment of an appropriate immune response. Eik Hoffmann (in Priscille Brodin's lab from Pasteur Lille, France) showed that mice lacking the enzyme IRG‐1, which regulates the production of the metabolite Itaconate, are not able to control *M. tuberculosis* infection. Interestingly, he showed that IRG‐1 is recruited to *M. tuberculosis*‐containing phagosomes and that this enzyme is required for the control of bacterial replication in macrophages. Immunometabolism is a field of growing interest, and knowing more about the role of such metabolites in infection may lead to novel alternatives to antibiotics.

Carrying on with the hijacked macrophage theme, the group of Marco Oggioni (University of Leicester) previously published that a single founder bacterium of *Streptococcus pneumoniae,* sequestered within splenic macrophages, can lead to sepsis after initial clearance in mice. Joe Wanford from this group wanted to see whether this was the case in humans. He reported an innovative organ‐slice perfusion model derived from experimentally infected ex vivo human spleens from patients undergoing elective splenectomy, which showed that intracellular bacteria accumulate with time post‐clearance, after a single monoclonal founder replicates to form a cluster. These data have obvious implications for how invasive pneumococcal disease should be treated.

Host cells are not always as vulnerable as they look, and cellular mechanisms that recognise and eliminate invasive bacterial pathogens are the subject of intense investigation. NRAMP1 (Natural Resistance‐Associated Macrophage Protein 1, also called Slc11a1) is a divalent‐metal efflux pump at the phagosomal membrane in macrophages, and mutations in NRAMP1 cause susceptibility to several intracellular pathogens. Using cutting edge single‐cell techniques, Olivier Cunrath (Dirk Bumann's lab at the Biozentrum, University of Basel, Switzerland) is looking to identify the precise mechanism of NRAMP1‐mediated resistance against *Salmonella* in vivo. Unexpectedly, he discovered that NRAMP1‐mediated resistance against infection is mediated by magnesium starvation, suggesting new strategies for infection control.

Previous work has shown that interferon exposure promotes cell‐autonomous immunity for pathogen control, but the underlying mechanisms are poorly understood. Michal Wandel (Felix Randow's lab at the^,^ MRC Laboratory for Molecular Biology) discovered that members of the interferon‐induced GTPase family of guanylate‐binding proteins (GBPs) control Gram‐negative bacteria that can invade the cytosol (e.g., *Salmonella* and *Shigella*). GBPs form a noncanonical inflammasome on the pathogen surface to initiate pyroptotic cell death of infected cells by recruiting and activating the cytosolic lipopolysaccharide receptor, Caspase‐4. Strikingly, *Shigella* can counteract GBP‐mediated cell‐autonomous immunity to inhibit cell death and form actin tails important for bacterial dissemination. As the next step, it will be of great interest to investigate the precise function for GBPs in host defence against cytosolic pathogens.

Ubiquitination of host and pathogen components defines much of the downstream signalling that regulates the recognition and control of intracellular pathogens. By using proteomics combined with the latex bead model of phagosomes, Tiaan Heunis (Matthias Trost's lab at the Newcastle University) showed us a holistic view of how proteins on phagosomes of activated macrophages are ubiquitinated. He demonstrated how immune activation qualitatively changes the phagosomal ubiquitin‐linkage landscape, identifying a plethora of novel targets that can potentially be modulated to target phagosomal pathogens. In fact, some of these ubiquitin‐modulating targets that regulate phagosome biology might be one day exploited as host‐directed therapies.

But the bacteria always seem to be one step ahead. Virtu Solano Collado (from Stefania Spano's lab at the University of Aberdeen) shared the latest update on her work investigating how Salmonella is able to subvert host trafficking pathways. Virtu described how *Salmonella typhimurium* effector proteins specifically target the small GTPase Rab32 to promote intracellular survival. She also presented new unpublished evidence of the importance of the Rab32/BLOC3 pathway in controlling *Salmonella* Typhi infection.

The field of cellular microbiology is exploding in part due to exciting advances in high resolution and high‐content microscopy. To quantify image‐based host‐pathogen interactions in an unbiased manner, Daniel Fisch (from Eva‐Maria Frickel's lab at the Francis Crick Institute) employed an artificial intelligence workflow to develop an image analysis platform called HRMAn (Host Response to Microbe Analysis, at https://hrman.org/). Using a variety of intracellular pathogens including the cellular microbiology paradigm *S*. Typhimurium, he showcased HRMAn's capacity to recognise and quantify various attributes of host‐pathogen interactions. In this way, HRMAn can inspire a broad range of cellular microbiologists to perform high‐content image analysis.

The final speaker of the day (kindly sponsored by *Cellular Microbiology*) was Thierry Soldati from the University of Geneva. Soldati—this journal's Editor‐in‐Chief—has pioneered the use of the amoeba *Dictyostelium discoideum* as a surrogate host for mycobacterial infections. In his keynote speech, he summarised some of his team's discoveries about how Mycobacterium marinum subvert and escape the phagosome, as well as spread between cells. He also described their most recent work showing, intriguingly, how *Dictyostelium* cells are able to exclude their infected brethren when they undergo multicellular development, effectively clearing the infection from the population.

In conclusion, the first meeting of the new Network was highly successful, exciting, and left everyone with much food for thought, along with new connections. And we liked it so much that we are doing it all over again in February 2020—this time in Sheffield. Please do join us!

